# Identification of the Parameters of the Szpica–Warakomski Method’s Rectilinear Trend Complementary to the Gaussian Characteristic Area Method in the Functional Evaluation of Gas Injectors

**DOI:** 10.3390/s25134020

**Published:** 2025-06-27

**Authors:** Dariusz Szpica, Jacek Hunicz, Andrzej Borawski, Grzegorz Mieczkowski, Paweł Woś, Bragadeshwaran Ashok

**Affiliations:** 1 Faculty of Mechanical Engineering, Bialystok University of Technology, 45 Wiejska Str., 15-351 Bialystok, Poland; a.borawski@pb.edu.pl (A.B.); g.mieczkowski@pb.edu.pl (G.M.); 2 Faculty of Mechanical Engineering, Lublin University of Technology, 36 Nadbystrzycka Str., 20-618 Lublin, Poland; j.hunicz@pollub.pl; 3 Faculty of Mechanical Engineering and Aeronautics, Rzeszow University of Technology, 12 Powstancow Warszawy Str., 35-959 Rzeszow, Poland; pwos@prz.edu.pl; 4 Department of Automotive Engineering, Vellore Institute of Technology, School of Mechanical Engineering, Vellore 632014, India; ashok.b@vit.ac.in

**Keywords:** combustion engines, alternative fuel supply, low-pressure gas-phase injector, research

## Abstract

The Fit for 55 and Euro 7 regulations significantly reduce CO_2_ emissions from combustion sources. This will be reflected in the regulations governing the approval of in-service vehicles, including those using alternative fuels. The present study focused on the rapid diagnostics of the technical condition of gas injectors. The test method was a modification of the Gaussian characteristic fields method using the Szpica–Warakomski rectilinear trend. The flow tests resulted in average volumetric intensities of 111 NL/min and 124 NL/min, depending on the operating conditions. The opening and closing times were in the range of (1.3…3.5) ms. The directional parameter of the rectilinear trend, which is important from the point of view of the analyses, was 0.97 for brand new (BN) injectors and 1.00 for in-service (IO) injectors. The intersection parameters were 0.64 and 0.24, respectively. The qualitative evaluation yielded coefficients of determination of 95.01 and 94.07. The values of the trend parameters were strongly dependent on the design solution and model/type of injector. Inferring the effect of operating condition on the trend parameter values, a one-factor analysis of variance was performed, which showed the significance of only the directional coefficient. A comparison of the same BN and IO injector model showed an apparent change in the value of the intercept only. No significant relationships between the injector opening and closing times and the trend parameters were shown. Thus, the usefulness of using the Szpica–Warakomski rectilinear trend in the functional evaluation of gas injectors of different designs and under different operating conditions was demonstrated.

## 1. Introduction

Modern legislation related to climate and energy policy mandates a reduction in CO_2_ emissions [1]. The largest emitters are listed as follows: EU, CN and US. They total about 50% of global emissions [2]. Globally, transportation accounts for 16% of global emissions and road transport for 12% [3], with the EU accounting for 27% and 19%, respectively [4,5]. New legislation being created for future years, such as Euro 7, LEV 4 and Low NOx, and is expected to reduce the permissible emission levels of NH_3_, N_2_O, CO, HC, NOx, and PM not only for engines powered by classical fuels but also hydrogen [6] in successive steps. Regulations relating to new species and particulate matter are also planned [7]. The climate agreement that was signed in Paris set a rise in the annual average temperature to just 1.5 °C by 2050. In addition to the widespread introduction of renewables, efforts are also focusing on CCTs [8]. The EU has recently introduced ‘Fit for 55′ regulations, significantly affecting the emissions performance of cars and vans [9,10]. As early as 1988, it was noted that the number of cars in the world exceeded 400 million and was 10 times higher than in 1950 [11], hence the increased legislative action on emissions. The year 2020 marks the 50th anniversary of the US Clean Air Act (EPA and NAAQS) for air pollutant criteria. Tackling increased emissions in transportation has resulted in various design and organizational solutions: EGR; TWC, SCR, DPF/FAP [7], S&S [12,13], ‘free-wheeling’ [14], car sharing and alternative means of transport [15,16], and ‘eco-driving’ [7]. There has been a steady increase in interest in alternative fuels, which must comply with CAFÉ and AMFA regulations [17,18]. New energy sources in vehicles (H_2_, FCV, BEV, MHEV, and PHEV) have not ruled out the use of ICE for the time being [19]. In particular, ICE’s GDIs using alternative fuels combined with assistance in hybrid systems have not exhausted their potential as sources of energy in transportation [20]. The use of oxygenated fuels, such as alcohols and their blends, can enhance combustion efficiency and reduce harmful exhaust emissions due to improved oxygen availability and cleaner burning characteristics [21]. Intensive research is being conducted on ethanol, butanol, and methanol for these benefits [21,22,23]. As a substitute, or in the form of mixtures, carbon-reduced fuels can be used in ICEs with different combustion organizations [24]. The most popular of this group are H_2_ [25], LPG [26,27], CNG [28,29], and LNG [30]. HVO [31], FAME [32], NH3 [33], and OME e-fuels [34] are also gaining popularity. A separate group includes fuels made from various types of waste or food products: biogas [35], syngas [25], PVO [36], GTL [37], biomass-gasifiers [38], POMDME [39], and TPO [40].

The most popular alternative fuels, which undoubtedly include LPG and CNG, today require low-pressure gas-phase injectors in their supply systems. Injectors of this type are normally closed solenoid valves that open for a specified period of time when electrical power is applied. Electromagnetic injectors are the most common in this field, although piezoelectric-driven injector concepts are being developed [41]. The injector is the last component of the power system and its role in the fuel system is decisive regarding the power supply and the formation of the combustible mixture.

In [42], the test methods applicable to the testing of injectors of various fuels in the main liquid fuels are presented in great detail, but the study of the issue of gas injectors and diagnostic methods is not exhaustive. For any power system, tests can be carried out on an engine dynamometer or chassis dynamometer using an exhaust gas analyzer [43,44], where a preliminary diagnosis of the fuel system will be possible. Among the most extensive and costly test methods are the Zeuch method [45]; Schlieren imaging method [46,47,48]; shadow classification and particle image accelerometry method [49]; high-speed cameras [50]; light fluorescence absorption [51]; optical lasers and laser absorption scattering techniques [52,53]; a combination of high-speed cameras and optical lasers [54]; X-ray [55]; heat flow sensors [56]; and long-range microscopy [57]. They allow a very detailed process evaluation but require a sophisticated apparatus and appropriate inference procedures. They are applicable to developmental research and the creation of new technical studies. As an alternative to these methods, there are simplified methods that allow the evaluation of basic functional parameters of importance in the initial diagnosis of the operating condition. Mainly, these are methods using lift sensors [58] and optical sensors [59].

The primary test method for diagnosing the operating condition of gas injectors is the determination of the volumetric flow rate using a flow meter [60]. Based on this, it is possible to evaluate the flow rate at fixed values of supply pressure and outlet nozzle diameter [61]. Using a surrogate fluid, the flow characteristics of a gas injector can also be determined, but the state of aggregation of the test medium can make the inference difficult [62]. An innovative approach in gas injector testing is presented in [63]. The ‘fuel tank refill’ method took into account the use of tanks and the test medium as air (often practiced for safety reasons). With this method, it was possible to simultaneously determine the flow characteristics of several injectors included in the array. As a result, the dosage irregularity of the gas rail was calculated. Some of the simplest diagnostic methods that can be used in the study of gas injectors are current and voltage in the power line [64]. These are indirect methods to determine the opening and closing times of injectors using waveforms from the power line (their curvature). Complementing this with acceleration sensors [65,66], the effectiveness of electrical measurements can be confirmed in a non-intrusive manner. An alternative to in-line electrical testing is presented in [67], where an injector nozzle outlet pressure sensor was used. The effectiveness of this method was confirmed for the lift sensor mounted in the injector under test. Tests using the pressure sensor allowed the evaluation of opening and closing times and the determination of dosing uniqueness, which is an obvious novelty in injector testing [67]. Condition verification using the methods presented earlier should be complemented by measurements of the electrical parameters of coils and circuits [68]. It should be noted at this point that the low-pressure gas-phase injectors do not have defined legal legislations, like, for example, gasoline injectors in SAE J1832 and J2715 [69].

One of the main problems in studying the low-pressure gas-phase injectors is access to comparative data. Manufacturers’ technical materials provide the maximum flow rate of continuously open injectors [70,71] and occasionally the flow characteristics of selected pressure values and outlet nozzle diameters [72,73]. Information on opening and closing times can also be found, but without specifying the method of determining them.

A literature analysis of the study of the low-pressure gas-phase injectors revealed a research gap in the area of rapid condition diagnostics. The research presented in this paper is a continuation of the analyses contained in [74]. The innovative Szpica–Warakomski (S-W) method presented there complemented the Gaussian characteristic area method used in diagnostics.

The main objective of this work was to determine the parameters of the rectilinear trend constituting the basis for inference in the S-W method for gas injectors of different designs and at different stages of operation.

The research question of this study was whether different designs and different operating conditions affect the indicators relevant for analyses using the Szpica–Warakomski method. In view of the research question formulated in this way, the determination of the rectilinear trend parameters underlying the S-W method for gas injectors of different designs and at different stages of operation was identified as the main objective. In addition, consistency, or the lack thereof, was sought in inferring the technical condition of gas injectors using the Gaussian characteristic figure area method and the Szpica–Warakomski rectilinear trend. The results obtained will be used in the future to determine the tolerance limits of the pattern.

The rest of the study is organized as follows: Section 2 presents the research objects. Section 3 describes the research methodology, equipment, and analysis methods. The research results and their discussion are included in Section 4, which was divided into the determination of the volumetric flow rate, opening and closing times, areas formed from measurements at significant points, and parameters of the rectilinear trend of the S-W method. The applicability of the results was also highlighted (Section 5). The entire study was summarized in Section 6, and future research in the subject area is finally described in Section 7.

The activities described in this manuscript revolve around the diagnosis of the condition of gas injectors, which is very important in the context of green transformation and the use of alternative fuels in internal combustion engines.

## 2. Subjects of This Research

The objects of this study were the low-pressure gas-phase injectors applicable to LPG and CNG vapor-phase supply systems. Injectors using a solenoid coil and movable opening elements in operation were selected for this study. Injectors of this type are normally closed solenoid injectors, in which, after the appearance of electrical power, the magnetic field of the coil causes them to open. In the analysis, different design solutions of injector valve actuators were considered in order to broaden the scope of inference. The objects of this study were injectors with valves opened by a piston, plunger, flap, and plate, as included in Table 1. In the plunger group, ‘c-f’ (cross-flow) was distinguished, in which the working medium, after passing through the control valve, does not flow in the axis of the valve, but bends at an angle of 90 deg.

## 3. Research Methods, Equipment, and Processing of Results

### 3.1. Research Methods

The research methods used in this study were aimed at determining the parameters of the rectilinear trend using the S-W method [74]. In order to look for possible reasons for the differences between the values obtained for different gas injectors, the scope of this study was extended to include the determination of functional parameters. In addition, the Gaussian half-surface method was included in the analysis of the results, which was juxtaposed with the S-W method. The scope of the research included

- Preliminary tests—a determination of the maximum volumetric flow rate *Q* and opening *t_o_* and closing *t_c_* times of injectors;
- Preliminary tests—a determination of Gaussian methods of the areas of the resulting geometric figures *A* built from the values *Q* obtained at different supply pressures *p* and injector opening times *t_imp_*;
- Main research—a determination of the parameters of the rectilinear trend of the S-W method on the basis of *Q* obtained at different *p* and *t_imp_*.

The first part of this study (preliminary research) was aimed at determining the basic functional characteristics of the injectors. Opening and closing times affect the volumetric flow rate when the injector is cycled. The maximum volumetric flow rate values given by injector manufacturers in technical documents [71,73,75] are determined with the injector constantly open. This does not directly translate into the flow characteristics of the injectors due to the different opening and closing times and the associated geometric characteristics of the valve elements. Therefore, it was considered important, in addition to determining the maximum volumetric flow rate, to determine these times. This was supported by their possible usefulness in further analyses when looking for sources of differences in the results of the main study.

The second part of the preliminary study is based on the descriptions presented in [76], where the half-Gaussian method was adapted to the functional evaluation of common-rail diesel injectors. A comparison of the fields of the resulting geometric figures determined at different supply pressures and opening times made it possible to predict the causes of injector failures. In the case of the low-pressure gas-phase injectors, as a novelty, the adoption of test points was necessary, bearing in mind that gas injectors basically operate at a single, stabilized fuel operating pressure.

The third stage of this research (the main research) is an original method developed by one of the authors of the study [74]. The method differs in its method of inference relative to the second part, while leaving the same research points. The innovation in this case is the replacement of the air field of the resulting geometric figure built from the research points by the parameters of the rectilinear trend obtained by linear regression. The parameters of the rectilinear trend, such as the directional parameter and the intersection parameter, are considered sufficient for evaluating the differences between injectors. In addition, the coefficient of determination shows the scatter of points with respect to the determined rectilinear trend.

### 3.2. Research Equipment

**Figure 1:**
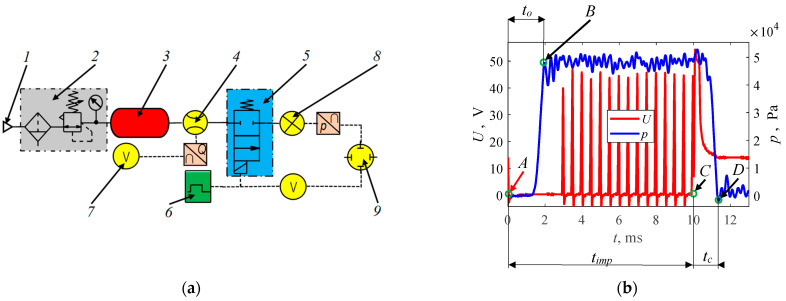
Schematic of the test stand: (**a**) *1*—air supply; *2*—air preparation system; *3*—buffer tank; *4*—mass flow meter; *5*—tested injector; *6*—injector control system; *7*—voltage meter; *8*—pressure sensor; and *9*—oscilloscope. Examples of the waveforms of electric supply voltage and pressure at the outlet of the injector nozzle (**b**).

The original test stand, shown in Figure 1a, was used to carry out the study. It is dedicated to flow testing of gas injectors, but can be used to test other solenoid valves at a specific differential pressure. Due to the danger of using the vapor phase of LPG or CNG, the tests were conducted on compressed air. This is a common procedure in gas injector testing [72,73,75]. Compressed air from air supply *1* flowed to air preparation system *2*, where it was subjected to cleaning and its pressure was established in the measuring system. Furthermore, the air went to buffer tank *3* with a volume of 40 L, which, in addition to accumulating the air supply, had the task of suppressing pulsations resulting from the cyclic operation of the injector. From buffer tank *3*, the air flowed through mass flow meter *4* to the injector *5* under test. In this case, a BRONKHORST F-113AC-M50-ABD-00-V flow meter using a thermal sensor and microprocessor-based signal conversion board in operation was used (response time 0.5 s; range (0…300) L_N_/min; output signal range (0…5) V; accuracy 0.5%). The control of the parameters of the tested injector was carried out using the injector control system (based on the STAG controller), allowing the control of the operating frequency, opening time, and PWM signal parameters responsible for sustained opening. The electrical voltage signal from the flow meter was read using an AXIOMET AX 102 voltage meter (range (200 m…600) V; accuracy ± 0.5%). With this bench configuration, it was possible to determine the maximum volumetric flow rate and conduct the second part of the preliminary and main tests. For the determination of the opening and closing times, the stand was supplemented with pressure sensor *8*. This sensor was the author’s solution described in [65,68], and the method of measurement and inference should be considered indirect, since it does not interfere with the actuator element of the injector valve. In operation, it used the MPXH6400A pressure transducer (response time <1 ms, range (20…400) kPa, output signal range (0…5) V, accuracy 0.25%) into which the air jet flowing out of the injector nozzle hit. Furthermore, the air jet left sensor *8* through the outlet holes into the atmosphere. Electrical signals from sensor *8* as well as from the injector’s electrical power line were transmitted to oscilloscope *9*. The oscilloscope used in the study was a RIGOL MSO4014 (bandwidth 100 MHz; real time sample rate up to 4 GSa/s; real time waveform record, replay, and analysis up to 200,000 frames; vertical resolution 8 bit).

### 3.3. Processing of Results

When measuring the maximum volumetric flow rate on the test stand (Figure 1a), a constant pressure was set in air preparation system *2* (1 × 10^5^ Pa) and the opening time of injector *5* was set large enough to cause continuous opening. At an operating frequency of 2000 imp/min, this was 30 ms, with a 95% filled PWM signal appearing after 3.5 ms to prevent overheating of the injector coil. The electrical voltage signal from flow meter *4* indicated on voltage meter *7* was converted according to the manufacturer’s data. In order to reduce noise during the measurement, a flexible tube with an inner diameter of 12 mm and a length of 5 m was installed at the outlet of the injector nozzle, thus taking the air out of the room in which the tests were conducted. It should be noted here that the gas injector was controlled by a short circuit to ground.

In determining the opening and closing times of the injectors, the test stand was supplemented with a pressure sensor *8* and an oscilloscope *9*. The pressure in the air preparation system *2* was set at (1 × 10^5^ Pa), the injection time at 10 ms, and the operating frequency at 1000 imp/min (corresponding to 2000 r./min for a 4-stroke engine). Having the waveforms of supply voltage and pressure at the outlet of the injector nozzle recorded with an oscilloscope, further data processing was performed using MATLAB 2023 software. The signals were limited with a 2nd-order Butterworth low-pass filter at a frequency of 0.25. Example waveforms are shown in Figure 1b. The opening time *t_o_* was determined from the onset of the control pulse *A* (in this case, shorted to ground) until the pressure at the injector nozzle outlet reached the first peak, indicating the maximum value *B*. The slight drop in pressure after the first maximum was reached was due to the reflection of the control element from the valve body. The closing time was determined from the disappearance of the control pulse *t_imp_* marked *C* to the pressure drop *D*. The correctness of this method has been confirmed in a number of publications, including [64,65,77]. Sometimes manufacturers specify the closing time only for the pressure drop phase after the disappearance of the electric pulse, which is not correct, since it is not referred to the beginning of the process *C*.

In the second part of the preliminary tests as well as in the main tests, the primary issue was to determine the test points for the supply pressure *p* and injection time *t_imp_*. For this purpose, the results of standardized [78,79] and non-standardized [80] tests of gas-fueled engines available in the literature were analyzed. In addition, a number of previous results of our own tests of engines with different power systems in daily use (data recorder AC STAG) were analyzed with a special emphasis on transients. Based on this, 5 test points were proposed, as shown in Table 2. Initially, the first test point defined the injection time at 2.0 ms, but preliminary tests on the test bench showed that a large proportion of the tested injectors were unable to overcome the supply pressure in this case, resulting in no flow (no injector opening).

**Figure 2:**
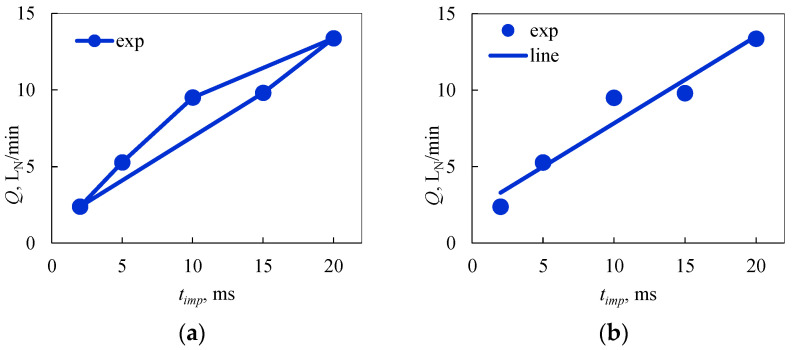
Determining the area of the characteristic figure (**a**) and a rectilinear trend from the measurement points (**b**).

The test points, in addition to their reference to the operating conditions of the supply system, have their rationale in diagnosing the condition of the injector. High pressure and short opening time (points 1 and 2) assess the operation of the electromagnetic system and changes in the characteristics of the compression spring (increase in stiffness caused by the presence of debris between the coils). A low pressure and long opening time (points 4 and 5) are diagnostics of the compression spring (increase in stiffness). Normal operation (point 3) provides diagnostics of changes in the stiffness of the compression spring and the presence of debris on the injector actuator. Details of the selection of test points (injection feed conditions) are presented in [74]. The case described in this study relates to LPG vapor-phase injectors. Based on this and using Table 2 in the second part of this study based on the results at the test points at each time, a geometric figure was created (Figure 2a). Test conditions at each point were established using air preparation system *3* and injector control system *6*.

The areas of the resulting geometric figures in the Cartesian coordinate system *Q* = f (*t_imp_*) based on its vertices were determined analytically using the Gaussian method [81]. Assuming that the vertices of each geometric figure in number i…n (*t_imp_*_1_, *Q*_1_), (*t_imp_*_2_, *Q*_2_),…, (*t_impn_*, *Q_n_*) were marked clockwise, the area was determined by the ‘shoelace method’ [82,83] of the general form

(1) $$A=0.5\left|\left(t_{imp1}Q_{2}+t_{imp2}Q_{3}+\ldots +t_{impn}Q_{1}\right)-\left(Q_{1}t_{imp2}+Q_{2}t_{imp3}+\ldots +Q_{n}t_{imp1}\right)\right|,$$

while taking into account the *t_imp_* coordination

(2) $$A_{1}=0.5\left|\sum_{i=1}^{n}t_{inji}\left(Q_{\left(i+1\right)}-Q_{\left(i-1\right)}\right)\right|,$$

and *Q_i_* coordination

(3) $$A_{2}=0.5\left|\sum_{i=1}^{n}Q_{i}\left(t_{imp(i+1)}-t_{imp(i-1)}\right)\right|.$$

The areas of *A*_1_ and *A*_2_ should give the same values.

In the main study, based on the results of measurements at points according to Table 2, a rectilinear trend was determined according to the S-W method using linear regression and the least squares method of deviations (Figure 2b).

The rectilinear trend was written as follows:

(4) $$Q=at_{imp}+b.$$

where its parameters were calculated from the relationship

(5) $$a=\frac{\sum \left(t_{imp}-{\bar{t}}_{imp}\right)\left(Q-\bar{Q}\right)}{\sum \left(t_{imp}-{\bar{t}}_{imp}\right)},$$

(6) $$b=\bar{Q}-a{\bar{t}}_{imp}.$$

A coefficient of determination was used to qualitatively assess the fit of the rectilinear trend to the points:

(7) $$R^{2}=100\;\frac{\sum \left(t_{imp}-{\bar{t}}_{imp}\right)\left(Q-\bar{Q}\right)}{\sqrt{{\sum \left(t_{imp}-{\bar{t}}_{imp}\right)}^{2}\sum {\left(Q-\bar{Q}\right)}^{2}}}.$$

where ${\bar{t}}_{imp}$ and $\bar{Q}$ are the arithmetic averages.

It was important from the cognitive point of view of this study to analyze the parameters of the rectilinear trend, such as the directional parameter and the intersection parameter in relation to the injectors, which are structurally different. An additional analysis was also aimed at confronting the values of the areas of the resulting geometric figures obtained from the measurement points with the values of the coefficients of determination. The novelty of the S-W method lies in its innovative inference of the state of the gas injector. As shown in [74], the use of the rectilinear trend of the S-W method has advantages over the Gaussian area field method. The area fields calculated according to the Gaussian method for injectors in different operating wear states can be similar, which does not allow for correct fault identification. It is particularly evident when the characteristic figures determined using this method were rotated. Using the rectilinear trend S-W method, it is possible to accurately identify the faults present using three parameters, such as the directional coefficient, the intercept, and the coefficient of determination.

## 4. Results and Discussion

### 4.1. Determination of the Maximum Volumetric Flow Rate and Injector Opening and Closing Times

**Figure 3:**
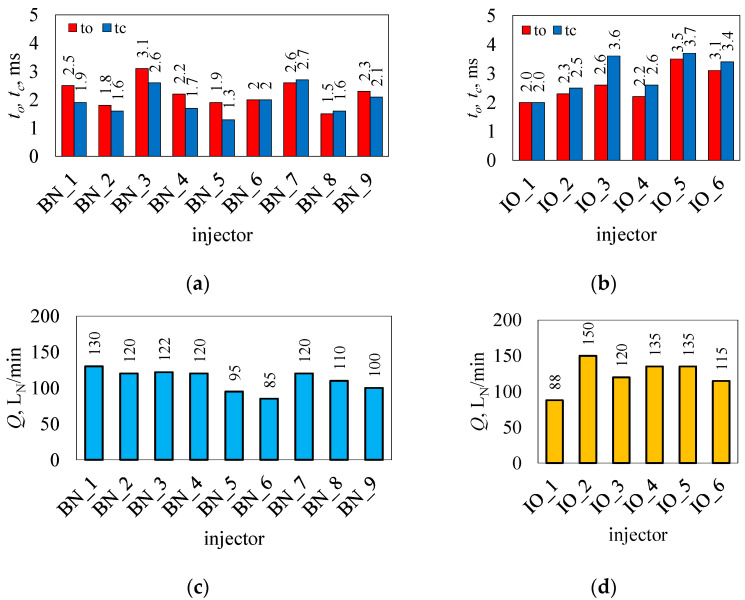
Opening (*t_o_*) and closing times (*t_c_*) of the tested injectors, brand new (**a**) and in operation (**b**), and maximum values of the volumetric flow rate of the tested injectors: brand new (**c**) and in operation (**d**).

In determining the volumetric flow rate *Q*, a constant air pressure of 1 × 10^5^ Pa was maintained in the measurement line. The injector was opened to the maximum with its opening sustained for 3.0 s, and the measurements were repeated five times. Due to the fact that the measurements concerned maximum values, nozzles with a maximum bore diameter (3 mm) were installed in the injectors, depending on their design, or they were tested without nozzles. In determining the opening and closing times, the air pressure in the measuring line did not change (1 × 10^5^ Pa), the opening pulse time was set at 10 ms, and the pulse frequency was set at 1000 imp/min. Figure 3 summarizes the results as average values from five times.

Of all the injectors tested (Figure 3a,b), as many as six showed an opening time *t_o_* equal to or greater than 2.5 ms (BN_1, BN_3, BN_7, IO_3, IO_5, and IO_6). This is important in the context of the first test point, according to Table 2. This can result in a worst-case scenario of no opening or reduced flow. The average opening time *t_o_* for brand new injectors was 2.2 ms, and that for injectors in operation was 2.6 ms. The closing times *t_c_* were 1.9 ms and 3.0 ms, respectively. The shortest opening time was shown by BN_8 (flap), it was 1.5 ms, while the longest time for IO_5 (piston) was 3.5 ms. The shortest closing time was recorded for BN_5 (plunger) at 1.3 ms, and the longest time for IO_5 (piston) was 3.7 ms. In the case of injectors labeled c-f (cross-flow), the indirect method test may be subject to error due to the collapse of the jet leaving the injector valve in the body. It was required for technical reasons that the opening and closing times be equal (*t_o_* = *t_c_*), which was noted for injectors BN_6 and IO_1. In such cases, there was a relationship between the time of the control pulse *t_imp_* and the response of the injector in the form *Q*. The worst in the relationship *t_o_*/*t_c_* was the IO_3 injector, and it was 2.6 ms/3.6 ms.

In the control systems for the operation of the gas engine supply system, in terms of the injectors used, very often manufacturers offer the possibility of selecting the type of injector from the level of the diagnostics and calibration program. The selection results in a change in the time after which the PWM signal appears. As the measurements showed, only the IO_5 injector with an opening time of 3.5 ms approached the limit of switching on the PWM signal in the adopted configuration of the injector control system used in the test bench. The other injectors tested showed opening times lower than 3.5 ms.

The results for *Q* are shown in Figure 3c, d. Due to the small scatter of results for the repetitions of each injector, the whiskers on the graph are not visible. The average value *Q* for brand new injectors was 111 L_N_/min, while for in-operation injectors, it was 124 L_N_/min. The higher average value for injectors in operation was due to the flow capacity of IO_2, which is an injector with increased output. The lowest value of *Q* was shown by injector BN_6 and was 85 L_N_/min. In contrast, the highest value of *Q* was that of the aforementioned IO_2, 150 L_N_/min. Depending on the needs of the required fuel supply conditions, the value of *Q* for a given engine type can be limited by the diameter of the nozzle mounted on the outlet of the injector (calibration nozzle). Nozzle diameters for the most part are nominally 1.5 mm; furthermore, in the machining process, they can be increased to about 3.0 mm. Not all injectors had the outlet nozzle fused to the body, in some cases it was necessary to mount them to the body. In the case of injectors with composite stubs, calibration nozzles were mounted internally. The name ‘calibration nozzle’ is associated with the process of calibrating the gas supply system to a particular engine type using dedicated computer software. In the case of calibration problems, a message in the form of ‘injector nozzle too small’ or ‘injector nozzle too large’ was often displayed. Analyzing Table 2 and Figure 3, there were no fundamental differences in *Q* depending on the design solution of the injector valve. The reason for the differences was the geometric parameters of the valve components, such as the diameters of the valve seats and the stroke of the injector moving element.

### 4.2. Determination of the Areas of Geometric Figures Constructed from Measuring Points

For the purpose of determining the areas of the resulting geometric figures using the Gaussian method, the tests were repeated five times at each of the measurement points specified in Table 2. Table A1 shows the average values of volumetric flow rate. Due to the scatter not exceeding 0.5 L_N_/min, the posting of deviations was abandoned. Despite an increase in the control pulse time at the shortest time tested from 2.0 ms to 2.5 ms at 2 × 10^5^ Pa, three of the injectors tested did not show flow at this point. Of the brand new injectors, it was BN_9, while of the injectors in operation, they were IO_5 and IO_6. In these cases, the electromagnetic force generated by the coil was unable to lift the flow control element. This was due to the significant pressure difference below and above the actuator and the associated hydraulic drag force. In these three cases, additional tests were carried out to demonstrate the maximum in-line pressure at which the injector would begin to open. The pressure was gradually lowered and the flow meter readings were observed. Tests with an opening pulse duration of 2.5 ms showed that injector BN_9 opened at 1.2 × 10^5^ Pa, while IO_5 opened at 1.1 × 10^5^ Pa and IO_6 opened at 0.7 × 10^5^ Pa.

**Figure 4:**
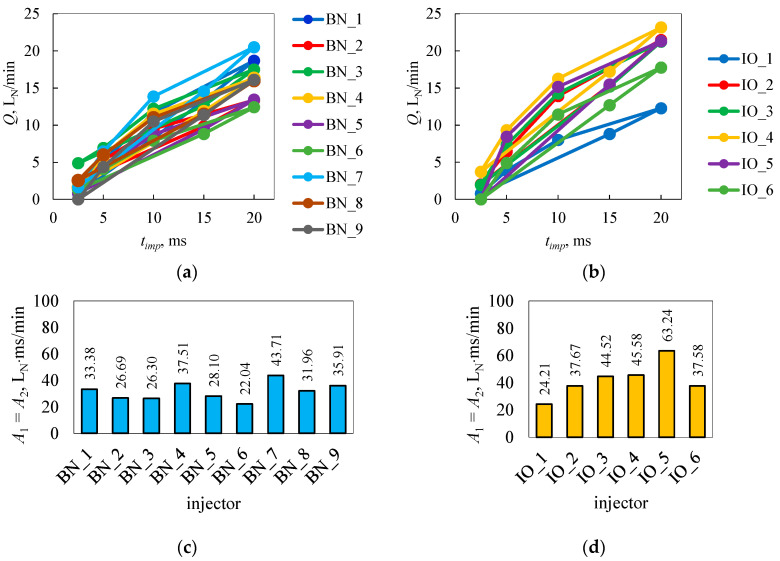
Geometric figures built from the research results of brand new (**a**) and in operation (**b**) injectors and surface areas of geometric figures of brand new (**c**) and in operation (**d**) injectors.

The resulting geometric figures from the measurement results contained in Table A1 are shown in Figure 4. The differences between the results at the different measurement points were so significant that the scatter whiskers at the test points were not visible.

Based on Table A1, the surface areas of the resulting figures were calculated using Equations (1) and (2), as shown in Figure 4a,b. The results of calculating the areas contained in Figure 4c,d showed variation in the values for individual injectors. The smallest area of the resulting geometric figure (22.04 L_N_ × ms/min) was calculated for injector BN_6, while the largest (63.24 L_N_ × ms/min) was calculated for IO_5. The average values of the areas of the resulting geometric figures are 36.73 L_N_ × ms/min for brand new injectors and 42.13 L_N_ × ms/min for injectors in operation. As can be seen in Figure 4c,d, brand new injectors show less variation in figure areas than those in operation.

The results in Figure 4a,b showed that the resulting figure could be similar in shape and therefore area, but was located above or below another figure being compared or rotated in some way relative to it. Therefore, the method using only the determination of the areas of the constructed figures did not give a clear result when making comparisons or searching for the source of injector defects. As an example here, BN_2 (26.69 L_N_ × ms/min) and BN_3 (26.30 L_N_ × ms/min) (Figure 4c,d) were rotated relative to each other. Hence, the idea of using another methodology in the inference came, which was the rectilinear trend. The parameters of the rectilinear trend are able to indicate the direction and position of the approximating line. In addition, the coefficient of determination evaluates the degree of clustering of points relative to the rectilinear trend. It was considered important to compare the values of the areas with the values of the coefficient of determination.

### 4.3. Determination of Rectilinear Trend Parameters Using the Szpica–Warakomski Method

**Figure 5:**
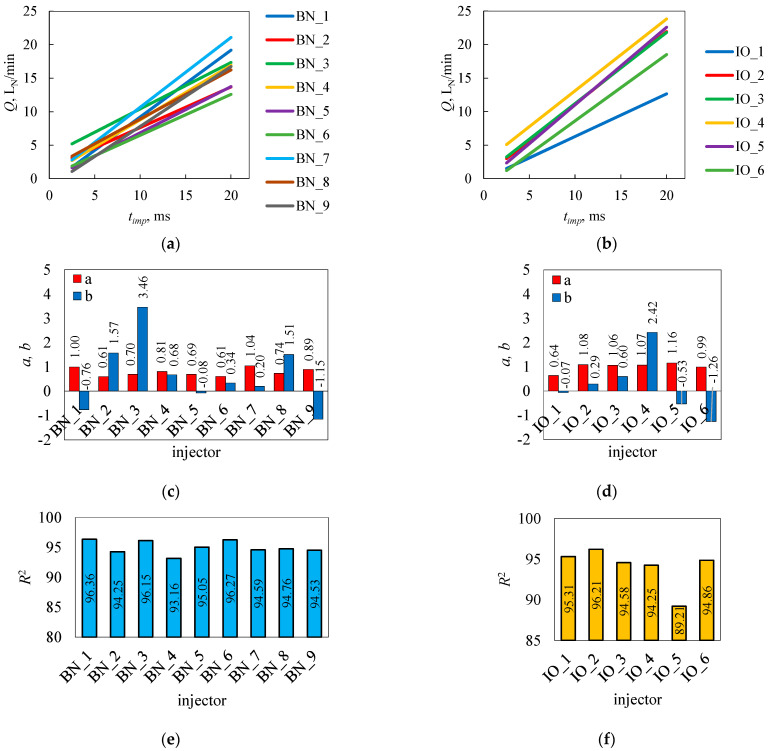
S-W method for the rectilinear trends for the tested injectors: brand new (**a**) and in operation (**b**). Parameters of the rectilinear trend: brand new (**c**) and in operation (**d**). Determination coefficients: brand new (**e**) and in operation (**f**).

Having the results presented in Table A1, rectilinear trends were determined using Equations (4)–(6) according to the S-W method (Figure 5). The location of the rectilinear trend for each injector varied, being more concentrated for brand new injectors than for injectors in operation. As shown in the previous section of this paper, the determination of the area created from the results of the geometric figure does not allow a precise assessment of the differences between injectors.

The values of the directional parameter of the rectilinear trend *a* for all tested injectors were in the range of (0.61…1.08) (Figure 5c,d). The average value *a* for brand new injectors was 0.79, and for injectors in operation, it was 1.00. The rectilinear trend intersection parameter *b* took both positive and negative values in the range (−1.26…+3.46). The average value *b* for brand new injectors was 0.64, while for those in operation, it was 0.24. For this rectilinear trend parameter, the differences may be due to the volumetric flow rate in cyclic operation, which does not necessarily relate to the value of maximum volumetric flow rate in continuous opening, which are shown in Figure 3c. The geometric parameters of the injector’s valve elements are important in this case. The coefficient of determination of the rectilinear trend *R*^2^ was in the range of (89.21…96.36). The spread *R*^2^, according to the assumptions of the S-W method, evaluated the clustering of the measurement points relative to the approximating rectilinear trend and could be reflected in the area of the geometric figure built from the measurement results.

Negative values of the intersection parameter *b* of the rectilinear trend were shown for BN_1, BN_5, BN_9, IO_1, IO_5, and IO_6. In the case of BN_9, IO_5, and IO_6, this may have been a consequence of the lack of flow at the first test point according to Table 2. Similarly, injectors BN_1, BN_5, and IO_1 showed a flow of less than 1 L_N_/min at this point, well below the measured values for the others that showed flow. The lack of opening at the first test point, according to Table 2 (2.5 ms), in addition to lowering the value *b*, can also cause changes in the value *a*. It was found that no or limited flow at the first test point can result in a value of parameter *b* below zero. The opposite situation was found for injector BN_3, which responded well to a high system pressure and short control pulse times. On the other hand, in the range of longer times, it had a problem with sustained opening. Injector IO_4 showed a highly localized rectilinear trend, with some of the highest index values.

The values of the coefficient of determination oscillated at 95.00 (Figure 5e,f). For brand new injectors, the average value *R*^2^ was 95.00. For injectors in operation, it was 94.00, with the IO_5 injector dominating this average result (89.21).

**Figure 6:**
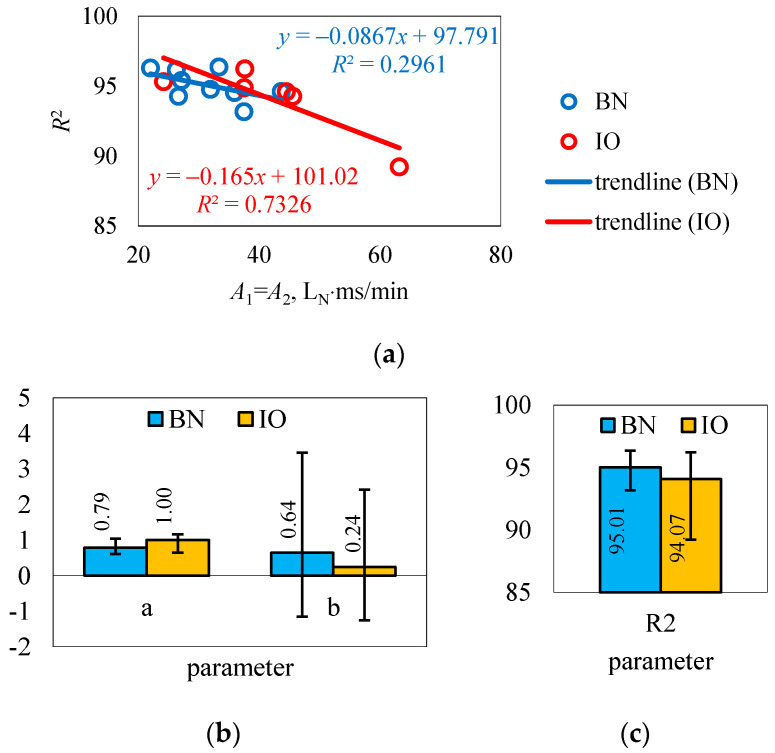
Correlation of determination coefficients with the areas of constructed figures (**a**) and values of the parameters of the rectilinear trend S-W method (**b**,**c**).

Correlations between the values of the areas of the figures formed from the test results of the points with the values of the coefficients of determination of the rectilinear trend of the S-W method were determined. As can be seen in Figure 6a, there was a correlation between the area fields and the coefficients of determination. A decrease in the value of the area field resulted in an increase in the coefficient of determination and vice versa, but this was not clear for the two groups compared (different directional parameters). Further work in this area is needed to confirm the assumption made.

In response to the main objective of this study, concerning the values of the parameters of the rectilinear trend S-W method, Figure 6b,c were created. In the studied group of brand new injectors, the value of the directional parameter was on average *a* = 0.79, with upper (+0.26) and lower (−0.18) deviations. This seems to correctly illustrate the average slope of the rectilinear trend of the survey and inference method in question. In the in operation group, the directional parameter *a* = 1.00 and is characterized by a slightly larger scatter relative to brand new injectors, amounting in the upper level of +0.16 and lower level of −0.36. The interval of the directional parameter (0.8…1.00) should be considered the reference for injector comparisons. The second significant parameter, which was the intersection parameter *b*, showed a very large variation, taking both positive and negative values. Brand new injectors gave an average value of *b* = 0.64, with upper (+2.82) and lower (−1.79) deviations, while injectors in operation had *b* = 0.24, with upper (+2.18) and lower (−1.50) deviations. In both cases, the deviations were multiples of the mean value. Such large discrepancies were due to the fact that the geometric parameters of the valve elements varied. This was not reflected in the maximum volumetric flow rate at continuous opening, and in cyclic flow, it turned out to be dominant. Therefore, the value of the parameter *b* should be determined in each case for a given type of injector, taking into account various factors, such as the diameter of the outlet nozzle, which limits the flow rate and, as a result, lowers the position of the rectilinear trend. The clustering of points with respect to the trend line assessed in the method in question by the coefficient of determination took average values for brand new injectors of *R*^2^ = 95.01 and for injectors in operation of *R*^2^ = 94.07. Deviations in this parameter were small, for brand new injectors the upper deviation was +1.35 and lower deviation was −1.85, and for injectors in operation, they were slightly larger, with an upper deviation of +2.14 and lower deviation of −4.85, but still represented a small percentage of the average value. In summary, the range of the coefficient of determination (94.00…95.00) should be considered representative at this stage of the study.

In response to the research question of this study, small differences in the mean values of the analyzed parameters of the rectilinear trend of the S-W method for injectors of different designs in both study groups were indicated (Figure 6b,c). It should be emphasized that this study was preliminary and conducted on small representative groups. On this basis, it was suggested to consider an individual approach to each design solution in comparative studies. With a view to assess the variability of the analyzed parameters of the S-W method during the operation of the injectors, it was decided to statistically assess the differences in the mean values based on the results from Figure 5c–f, taking into account the different numbers in the groups. A one-way ANOVA (Excel) was carried out, with the null hypothesis *H*_0_ stating the equality of mean values of the analyzed parameters and the alternative hypothesis *H*_1_ rejecting this equality. The results are presented in Table 3.

The variance values (Table 3) confirm the variation in each of the analyzed parameters (*a*, *b*, and *R*^2^), depending on the BN and IO groups. The table also includes values for the sum of squares of deviations *SS*, the number of degrees of freedom *df*, and the mean square error *MS* within groups (columns) and between groups (error).

The *F* value was the value of the statistic that we refer to *Test F*, while *p* was the probability. Assuming a confidence level of 0.05, was found that only for parameter *a* a probability *p* of 0.033 confirmed the rejection of hypothesis *H*_0_ in favor of hypothesis *H*_1_ in the analysis of the influence of operating conditions. In this case, the mean values differed significantly. This was confirmed by the *F* value of 5.66, which was above the 4.67 resulting from the *Test F*. In the case of the other two parameters, *b* and *R*^2^, the values of the statistics were above 0.05 and were 0.58 and 0.31, respectively. This indicates that there was no basis for rejecting the *H*_0_ hypothesis of the equality of mean values. With this in mind, it was concluded that the decisive parameter for the comparative study of both injectors in the BN and IO groups and between groups would be the directional coefficient of the straight line *a*. Other parameters, such as the intercept *b* and the coefficient of determination *R*^2^, would not be statistically significantly different. In the case of *R*^2^, the variance value in the IO group was significantly influenced by injector IO_5 with a value of 89.21, where the others oscillated in the range (94…96). This confirms the need for an individual approach to the study of individual injector models. It was considered reasonable to establish the S-W rectilinear trend parameters for individual brand new models and to provide tolerance limits for injectors in service. The results of this study form the basis for determining the tolerance limits based on a group with varying conditions and technical solutions.

**Figure 7:**
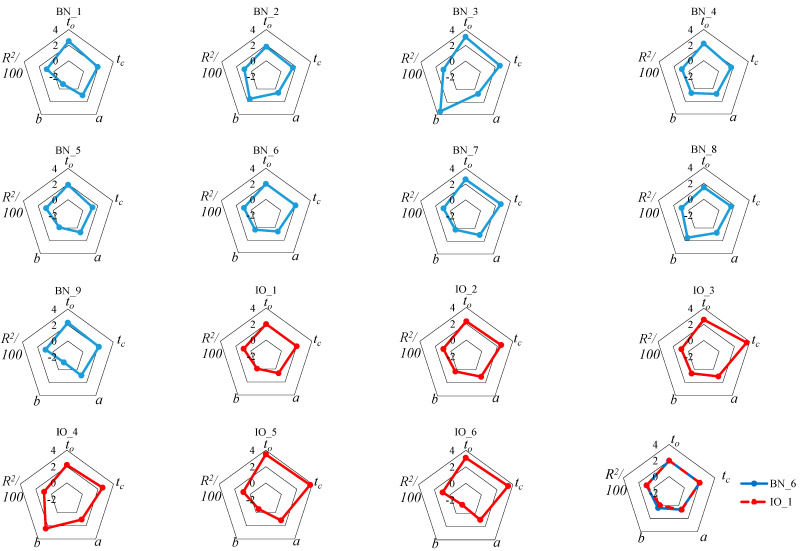
Radar charts with values of the coefficients relevant to the analysis of the tested injectors.

Due to the fact that there was some novelty in the analyses associated with the S-W method, there was a lack of data for comparisons with the results of this study. Therefore, it was decided to compile the results of this study into radar plots in order to further search for reasons for the differences between the values of the parameters of the rectilinear trend for the injectors studied. To achieve this, the coefficient of determination *R*^2^/100 was used, and the scale of the graph was set at (−2…4) (Figure 7). In the search for the relationship of the values of *a*, *b*, and *R*^2^ with the opening time *t_o_* and closing time *t_c_*, a lack of correlation with respect to the parameter *b* in particular became apparent. Both the short opening and closing times of brand new BN_1 or BN_4 injectors gave different values of *b*. The variation in *b* was best shown by comparing BN_3 and BN_9. For injectors in operation, the opening *t_o_* and closing *t_c_* times, except for IO_1, were longer than in brand new injectors. As before, this yielded different values of *b*. Parameters *a* and *R*^2^ did not have as much variation as *b*, but should be determined on a case-by-case basis for individual injectors. It does not directly follow from Figure 7 that the values of *a* and *R*^2^ depend on the design solution of the injector valve. Parameter *b*, on the other hand, reflects the geometric characteristics of the injector valve, and so it should be determined for comparisons on a case-by-case basis.

Having established the fact that the significant parameters of the S-W method rectilinear trend can be averaged only in terms of the directional parameter and the coefficient of determination, while the intersection parameter should depend on the geometric parameters of the valve element, a comparison was made between two injectors from one manufacturer, which were BN_6 and IO_1 (Figure 7). In spite of the almost 10-year difference in production date separating them and, in the case of IO_1, a vehicle mileage of about 80,000 km, there was apparent agreement in the values of the parameters *t_o_*, *t_c_*, *a*, and *R*^2^. There was a slight difference for parameter *b*, which may be due to the different designs of the injector valve system components. This once again confirms the need to determine parameter *b* individually for each injector.

## 5. Application Guidelines

The measurement and analysis results presented in this paper allowed the determination of the average values of the significant parameters of the rectilinear trend of the S-W method. The directional and determination parameters showed acceptable scatter with respect to the average, while the point of intersection resulted from the geometric parameters of the design solution of the valve elements. Therefore, it seems most authoritative to use the S-W method in the preliminary diagnosis of the technical condition of gas injectors, especially those placed in a common rail. In this way, it will be possible, having a standard, of course, to compare injectors of the same design among themselves and direct them to calibration (brand new) or regeneration (in operation).

## 6. Conclusions

In the course of the conducted activities, nine brand new injectors and six injectors in operation from the group of the low-pressure gas-phase injectors were analyzed using the original test stand and the adopted innovative inference, and the following conclusions were drawn:

1. Of all the injectors tested, six showed an opening time *t_o_* equal to or greater than 2.5 ms (BN_1, BN_3, BN_7, IO_3, IO_5, and IO_6), which affected the subsequent analysis of the results.
2. The average value of volumetric flow rate *Q* for brand new injectors was 111 L_N_/min and for those in operation was 124 L_N_/min. The lowest value *Q* was obtained for injector BN_6 (85 L_N_/min) and the highest for IO_2 (150 L_N_/min).
3. The average opening time *t_o_* for brand new injectors was 2.2 ms and for injectors in operation was 2.6 ms. Closing times *t_c_* were 1.9 ms and 3.0 ms, respectively. The shortest opening time was shown for BN_8 (1.5 ms), while the longest was shown for IO_5 (3.5 ms). The shortest closing time was shown for BN_5 (1.3 ms) and the longest for IO_5 (3.7 ms). The worst in the relationship (*t_o_*/*t_c_*) was the IO_3 injector; it was 2.6 ms/3.6 ms.
4. In the proposed innovative test method (S-W), at the point characterized by the shortest opening time and highest pressure, in three cases, the injectors did not open. Gradual lowering of pressure showed opening for BN_9 at 1.2 × 10^5^ Pa, IO_5 at 1.1 × 10^5^ Pa, and IO_6 at 0.7 × 10^5^ Pa.
5. The smallest area of the geometric figure formed from the results of the test points (22.04 L_N_ × ms/min) was calculated for injector BN_6 and the largest (63.24 L_N_ × ms/min) for IO_5. The average values were 36.73 L_N_ × ms/min for brand new injectors and 42.13 L_N_ × ms/min for injectors in operation. The method using only the determination of the area of the constructed figures to infer the state of the injector may not give a clear result when comparing, as an example, BN_2 (26.69 L_N_ × ms/min) and BN_3 (26.30 L_N_ × ms/min), which are rotated relative to each other.
6. The values of the directional parameter of the rectilinear trend of the S-W method averaged *a* = 0.79 with upper (+0.26) and lower (−0.18) deviations. In the in operation group, the parameter *a* = 1.00 and was characterized by a slightly larger scatter relative to brand new injectors, amounting in the upper level of +0.16 and lower level of −0.36. The interval of the directional parameter (0.8…1.00) should be considered a reference for injector comparisons.
7. The parameter of the intersection of the rectilinear trend of the S-W method showed a very large variation, taking both positive and negative values. Brand new injectors gave an average value *b* = 0.64, with upper (+2.82) and lower (−1.79) deviations. In injectors in operation, *b* = 0.24, with upper (+2.18) and lower (−1.50) deviations. In both cases, the deviations were multiples of the average value. Such large differences were due to the fact that the geometric parameters of the injector’s valve elements varied. Therefore, the value of the coefficient *b* should be determined in each case for a given type of injector, taking into account various factors, such as the diameter of the outlet nozzle, which restricts the flow and, as a result, lowers the position of the rectilinear trend.
8. The coefficient of determination of the rectilinear trend of the S-W method took average values for brand new injectors *R*^2^ = 95.01 and for injectors in operation *R*^2^ = 94.07. The deviations for this parameter were small; for brand new injections the upper deviation was +1.35 and lower deviation was −1.85, and for injectors in operation, they were slightly larger, upper (+2.14) and lower (−4.85), but still represented a small percentage of the average value. In summary, the range of the coefficient of determination 94…95 should be considered as a reference at this stage of this research.
9. The one-way analysis of variance to assess the significance of differences in the mean values of the S-W rectilinear trend parameters for the BN and IO cases yielded a positive result only for the directional coefficient *a*. The intercept and the coefficient of determination reached probabilities well above the accepted threshold of 0.05, indicating that there were no grounds for rejecting the hypothesis that the mean values of the BN and IO groups were equal. This showed that only the directional coefficient was affected by the operating time.
10. The summary of test results in radar charts showed no direct relationship of parameters *a*, *b*, and *R*^2^ with the opening time *t_o_* and closing time *t_c_*. The values of *a* and *R*^2^ clearly did not depend on the design solution of the injector valve. Parameter *b*, on the other hand, reflected the geometric characteristics of the injector valve, and so it should be determined for comparisons individually.
11. The research presented in this study was preliminary and needs to be gradually supplemented with analyses of injectors of the same type under different operating conditions. However, based on the results of this study, it was found that injectors of different designs and in different operating conditions gave varying values of the S-W rectilinear trend parameters. In one comparison, where the same injector model was confronted with each other, the differences for the BN and IO cases were mainly related to the intersection point *b*. This means that the exploitation influences the vertical shift of the trend line.

## 7. Further Research

In further activities, it was planned to carry out tests of injectors with the same design solutions to determine the tolerance limits of the parameters of the rectilinear trend of the S-W method for the selected manufacturer and model. It was also planned to develop a concept for the design of a test stand implementing tests in automatic mode with the possibility of comparing to a standard and inferring the potential source of injector deficiency.

## Figures and Tables

**Table 1:** Technical data of the injectors.

**Injector**	**Valve Type**	**Coil Resistance Ω**	** 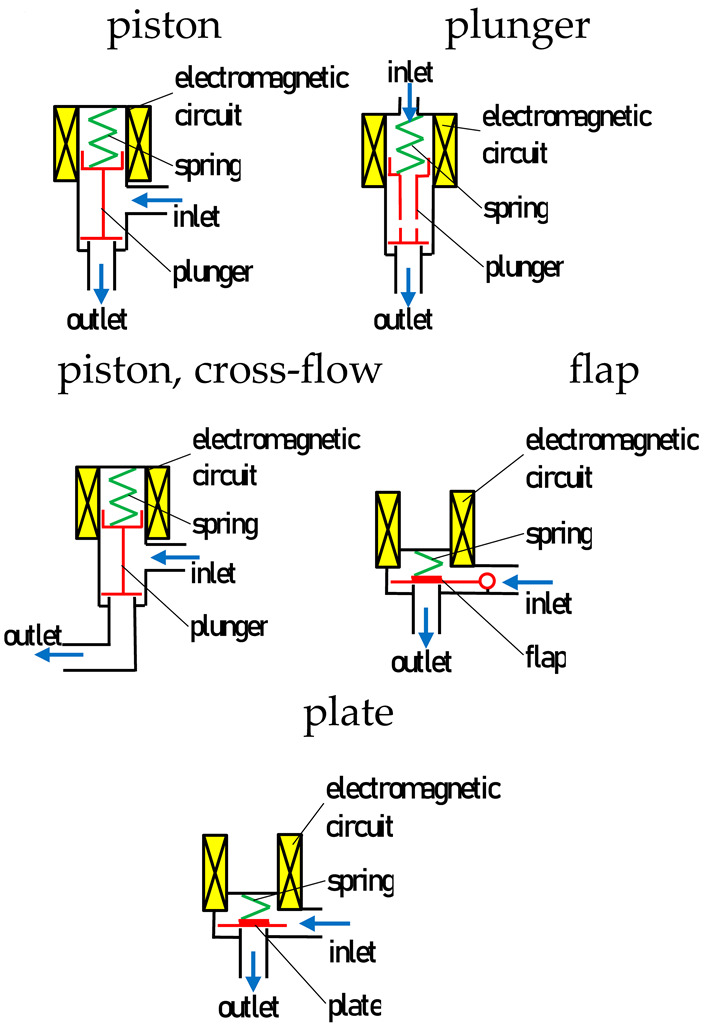 **
**brand new (BN)**			
AC-W01-4	piston	2.00	
Valtek 39STD	piston	2.50	
TOMASETTO Achille IT01	piston	2.00	
ALEX Barracuda	plunger	1.90	
HANA H2000 Red	plunger	1.90	
KEIHIN Blue	plunger	1.25	
OMVL Dream XXI SL	piston, c-f	3.00	
MATRIX HSF.211.20	flap	2.00	
ACON Apis Solo	plate	2.00	
**in operation (IO)**			
KEIHIN Blue	plunger	1.25	
MAGIC JET	piston	2.00	
OMVL REG Fast Black	piston, c-f	3.00	
KME IG3 Horizon	piston	2.80	
ELPGAS IG1 Stella VERDE	piston	3.00	
MWM FOCUS	piston	3.00	

**Table 2:** Test point supply pressures and opening times.

No.	*p*, 10^5^ Pa	*t_inj_*, ms	Operating States
1	2	2.5	start of operation of the LPG system after switching over to gaseous fuels
2	1.5	5	further operation after switching over to LPG
3	1	10	normal operation of the LPG system
4	0.5	15	insufficient evaporator outlet (naturally aspirated engine)
5	0.5	20	insufficient evaporator outlet (supercharged engine)

**Table 3:** Results of one-way ANOVA.

Group	Number	Average/Variance				
		*a*	*b*	*R* ^2^		
Brand New (BN)	9	0.79/0.0253	0.64/1.9464	95.01/1.1627		
In Operation (IO)	6	1.00/0.0340	0.24/1.5647	94.07/6.1307		
**analysis of variance**						
Source	*SS*	*df*	*MS*	*F*	*p*	*Test F*
**directional parameter *a***						
Columns	0.1621	1	0.1621	5.6577	0.0334	4.6672
Error	0.3726	13	0.0287			
Total	0.5347	14				
**intersection parameter *b***						
Columns	0.5680	1	0.5680	0.3156	0.5838	4.6672
Error	23.3946	13	1.7996			
Total	23.9626	14				
**coefficient of determination *R*^2^**						
Columns	3.4574	1	3.4574	1.1249	0.3082	4.6672
Error	39.9550	13	3.0735			
Total	43.4124	14				

## Data Availability

The datasets generated during and/or analyzed during the current study are available from the corresponding author upon reasonable request.

## Abbreviations

The following abbreviations are used in this manuscript:

AMFA	Alternative Motor Fuels Act
BEV	Battery Electric Vehicle
CAFÉ	Corporate Average Fuel Economy
car sharing	The model of car rental where people rent cars for short periods of time, often by the hour
CARB	California Air Resources Board
CCT	Carbon Credit Token
CN	China
CNG	Compressed Natural Gas
CO	Carbon Monoxide
CO_2_	Carbon Dioxide
DPF/FAP	Diesel Particulate Filter
eco-driving	Driving a car that allows the lowest possible fuel consumption
EGR	Exhaust Gas Recirculation
EPA	Environmental Protection Agency
EU	European Union
EURO 7	European Vehicle Emissions Standards
FAME	Fatty Acid Methyl Ester
FCV	Fuel-Cell Electric Hybrid Vehicle
free-wheeling	The mechanism that allows the vehicle’s wheels to rotate freely without the need for engine power
GTL	Gas to Liquid
H_2_	Hydrogen
HC	Hydrocarbon
HVO	Hydrotreated Vegetable Oil
ICE	Internal Combustion Engine
LEV 4	Low-Emission Vehicle
LNG	Liquefied Natural Gas
Low NOx	Key Elements of EPA’s 2027 Low-NOx Rule
LPG	Liquefied Petroleum Gas
MHEV	Mild Hybrid Electric Vehicle
N_2_O, NOx	Nitrous Oxide
NAAQS	National Ambient Air Quality Standards
NH_3_	Ammonia
OME	Oxymethylene Ether
PHEV	Plug-in Hybrid Electric Vehicle
PM	Particulate Matter
POMDME	Polyoxymethylene Dimethyl Ether
PVO	Pure Vegetable Oil
S&S	Start and Stop
SCR	Selective Catalytic Reduction
S-W	Szpica–Warakomski method
TPO	Tire Pyrolytic Oil
TWC	Three-Way Catalyst; US, United States of America
*A*, *A*_1_, and *A*_2_	Polygon surface area
*a*	Slope
*b*	Intersection
*p*	Pressure
*Q*	Volumetric flow rate
*R*^2^	Coefficient of determination
*t_c_*	Closing time
*t_inj_*	Injection time
*t_o_*	Opening time

## Appendix A

**Table A1:** Volumetric flow rate values determined at the measurement points.

Code	Valve Type	Injector	2.5 ms, 2 × 10^5^ Pa	5 ms, 1.5 × 10^5^ Pa	10 ms, 1 × 10^5^ Pa	20 ms, 0.5 × 10^5^ Pa	15 ms, 0.5 × 10^5^ Pa
brand new (BN)—L_N_/min							
BN_1	piston	AC-W01-4	0.78	5.15	11.65	18.68	13.27
BN_2	piston	VALTEK 39STD	2.38	5.27	9.50	13.37	9.82
BN_3	piston	TOMASETTO Achille IT01	4.87	6.92	12.19	17.50	12.62
BN_4	plunger	ALEX Barracuda	1.58	5.91	11.48	16.31	11.84
BN_5	plunger	HANA H2000 Red	0.86	4.23	8.63	13.43	9.45
BN_6	plunger	KEIHIN Blue	1.28	4.07	7.91	12.41	8.81
BN_7	piston, c-f	OMVL Dream XXI SL	1.64	6.45	13.85	20.50	14.56
BN_8	flap	MATRIX HSF.211.20	2.56	5.99	11.02	15.99	11.47
BN_9	plate	ACON Apis Solo	0.00	4.36	10.49	16.09	11.36
in operation (IO)—L_N_/min							
IO_1	plunger	KEIHIN Blue	0.80	3.88	8.00	12.28	8.81
IO_2	piston	MAGIC JET	2.03	6.47	13.93	21.46	15.37
IO_3	piston, c-f	OMVL REG Fast Black	1.96	7.33	14.26	21.24	15.22
IO_4	piston	KME IG3 Horizon	3.71	9.31	16.26	23.16	17.21
IO_5	piston	ELPGAS IG1 Stella VERDE	0.00	8.44	15.16	21.31	15.47
IO_6	piston	MWM FOCUS	0.00	4.92	11.44	17.76	12.69
